# Comparison between regional citrate anticoagulation and heparin for intermittent hemodialysis in ICU patients: a propensity score-matched cohort study

**DOI:** 10.1186/s13613-021-00803-x

**Published:** 2021-01-22

**Authors:** Christophe Leroy, Bruno Pereira, Edouard Soum, Claire Bachelier, Elisabeth Coupez, Laure Calvet, Konstantinos Bachoumas, Claire Dupuis, Bertrand Souweine, Alexandre Lautrette

**Affiliations:** 1 Medical Intensive Care Unit, Gabriel-Montpied University Hospital, Clermont-Ferrand, France; 2 Intensive Care Unit, Regional Hospital Center, Puy en Velay, France; 3 Biostatistics Unit (DRCI), Gabriel-Montpied University Hospital, Clermont-Ferrand, France; 4 LMGE (Laboratoire Micro-Organismes: Génome et Environnement), UMR CNRS 6023, Université Clermont Auvergne, Clermont-Ferrand, France; 5 Intensive Care Medicine, Gabriel Montpied Teaching Hospital, Intensive Care Unit, Centre Jean Perrin, 54 rue Montalembert, BP69, 63003 Clermont-Ferrand, Cedex 1, France

**Keywords:** Intermittent hemodialysis, Regional citrate anticoagulation, ICU, Heparin anticoagulation, Renal replacement therapy

## Abstract

**Background:**

Regional citrate anticoagulation (RCA) is the gold standard of anticoagulation for continuous renal replacement therapy but is rarely used for intermittent hemodialysis (IHD) in ICU. Few studies assessed the safety and efficacy of RCA during IHD in ICU; however, no data are available comparing RCA to heparin anticoagulation, which are commonly used for IHD. The aim of this study was to assess the efficacy and safety of RCA compared to heparin anticoagulation during IHD.

**Methods:**

This retrospective single-center cohort study included consecutive ICU patients treated with either heparin anticoagulation (unfractionated or low-molecular-weight heparin) or RCA for IHD from July to September in 2015 and 2017. RCA was performed with citrate infusion according to blood flow and calcium infusion by diffusive influx from dialysate. Using a propensity score analysis, as the primary endpoint we assessed whether RCA improved efficacy, quantified with Kt/V from the ionic dialysance, compared to heparin anticoagulation. The secondary endpoint was safety. Exploratory analyses were performed on the changes in efficacy and safety between the implementation period (2015) and at long term (2017).

**Results:**

In total, 208 IHD sessions were performed in 56 patients and were compared (124 RCA and 84 heparin coagulation). There was no difference in Kt/V between RCA and heparin (0.95 ± 0.38 vs. 0.89 ± 0.32; *p* = 0.98). A higher number of circuit clotting (12.9% vs. 2.4%; *p* = 0.02) and premature interruption resulting from acute high transmembrane pressure (21% vs. 7%; *p* = 0.02) occurred in the RCA sessions compared to the heparin sessions. In the propensity score-matching analysis, RCA was associated with an increased risk of circuit clotting (absolute differences = 0.10, 95% CI [0.03–0.18]; *p* = 0.008). There was no difference in efficacy and safety between the two time periods (2015 and 2017).

**Conclusion:**

RCA with calcium infusion by diffusive influx from dialysate for IHD was easy to implement with stable long-term efficacy and safety but did not improve efficacy and could be associated with an increased risk of circuit clotting compared to heparin anticoagulation in non-selected ICU patients. Randomized trials to determine the best anticoagulation for IHD in ICU patients should be conducted in a variety of settings.

## Background

Renal replacement therapy (RRT) is required for up to one-fifth of patients with acute kidney injury admitted to an intensive care unit (ICU) [1]. Worldwide, the majority of RRT procedures in ICU is performed with a continuous renal replacement therapy (CRRT), but intermittent hemodialysis (IHD) is used in 25% of cases [1, 2].

Anticoagulation is required for RRT to prevent clotting of the extracorporeal circuit [3]. Systemic heparin anticoagulation is commonly used during IHD but increases the risk of bleeding that raises ICU mortality [4]. A heparin-free RRT anticoagulation strategy is needed for patients with a high bleeding risk, including those with active bleeding, recent surgery, or those with heparin-induced thrombocytopenia. In the 1990s, regional citrate anticoagulation (RCA) was developed for CRRT [5]. Several studies showed its safety and efficacy [6, 7] which lead to the 2012 guidelines of the Kidney Disease Improving Global Outcomes, recommending RCA rather than heparin anticoagulation in patients treated with CRRT [8]. In comparison to heparin anticoagulation during CRRT, RCA is shown to reduce the risk of circuit clotting, the bio-incompatibility related to inflammation, and may improve the survival [9–11]. RCA for CRRT is currently easily controlled and thus widely used in ICU. On the other hand, RCA for IHD is more complex to use because of the elevated rates of blood flow and dialysate requires high citrate flow leading to an increased risk of sudden and fatal hypocalcemia [12].

Recent pilot studies have reported safety and efficacy of RCA during IHD sessions in ICU patients [13–16]; however, no data comparing RCA to heparin anticoagulation have been available. Additionally, only a few ICU departments use RCA during IHD in routine due to its high complexity. As the primary endpoint, we assessed whether RCA improved efficacy, quantified with Kt/V from the ionic dialysance at the end of IHD session, compared to heparin anticoagulation during IHD in ICU patients. The secondary endpoint was to compare safety. Exploratory analyses were performed on the changes in efficacy and safety between an implementation period (2015) and at long term (2017).

## Materials and methods

### Study design

This study was a retrospective cohort study, including all consecutive patients treated with either heparin anticoagulation (unfractionated or low-molecular-weight heparin) or RCA for IHD and admitted to the medical ICU of the Clermont-Ferrand University Hospital in France between 1 July and 30 September in 2015 and 2017. Patients were identified using the French national hospital database (Program for Medicalization of Information Systems) with the International Classification of Diseases diagnostic codes for “acute renal failure” and “chronic renal failure” and the therapy code for “renal replacement therapy.” Exclusion criteria were the use of Danaparoid anticoagulation for IHD, the absence of an anticoagulation during IHD, the ultrafiltration sessions without hemodialysis, and if there were missing data in electronic medical record regarding the primary objective. Patients were notified during their hospital stay that data would be abstracted from their medical record and used for research purposes. This study was approved by the Comité de Protection des Personnes Sud-Est 6 (reference # 2015/CE72–NoIRB00008526) and was reported according to the STROBE guidelines regarding observational cohort studies.

### Intermittent hemodialysis and anticoagulation procedures

IHD sessions were performed using AK200 (Gambro®) generators and polysulfone high-flux membranes (Hemotech®). The RCA during IHD was performed according to the procedure previously described [14, 17, 18]. Sodium Citrate 4% (Fresenius®) was infused immediately downstream from the connection between the dialysis catheter and arterial line of the circuit (Additional file 1: Figure S1). The citrate flow was set according to blood flow to reach a citrate blood concentration between 3 and 4 mmol/L, resulting in a blood ionized calcium level lower than 0.4 mmol/L to achieve an effective anticoagulation before the dialysis membrane, as proposed in CRRT [19]. Calcium supplementation was achieved with the diffusion of calcium contained in the dialysate. The calcium concentration of the dialysate was either 1.25 mmol/L or 1.5 mmol/L according to the patient's ionized calcium level at the beginning of a session being higher or lower than 1.15 mmol/L, respectively. Monitoring of a patient's ionized calcium was performed at least one hour after and at the end of a session. Calcium gluconate 10% infusion was added when the patient's ionized calcium level was lower than 0.90 mmol/L. The volume of citrate solution infused during the session was added to the prescribed ultrafiltration. A written protocol guided intensivists in prescribing the citrate flow rate and in selecting the calcium concentration of the dialysate, based on the prescribed blood flow and patient's ionized calcium level. RCA for IHD was implemented on February 2015 in our ICU and was entirely controlled in routine clinical practice from the beginning of the first study period.

Our protocol on heparin anticoagulation during IHD was in accordance with standard practice [8] and recommended either as an injection of low-molecular-weight heparin (Enoxaparin 40 mg) at the start of a session or with a bolus (50 U/kg) followed by a continuous infusion of unfractionated heparin (15 U/kg/h) during the session. If a patient was already treated with a continuous infusion of unfractionated heparin, then the same anticoagulation was used during IHD session with the same dose. All intensivists were received specific training twice a year by a nephrologist–intensivist on IHD according to national guidelines [20], especially anticoagulation procedures, and they controlled both anticoagulation procedures. The choice of anticoagulation for IHD was left to the discretion of the providing intensivists.

### Data collection

Patient and IHD characteristics were extracted from the patient’s electronic medical record. Patient characteristics included age, sex, weight and body mass index (BMI) at ICU admission, end-stage kidney failure requiring RRT, simplified acute physiology score (SAPS) II at ICU admission, sepsis in ICU stay, invasive mechanical ventilation, and vasopressor. IHD characteristics included the location and type of vascular access, anticoagulation type (RCA or heparin), contraindication to heparin anticoagulation defined as active bleeding or heparin-induced thrombocytopenia, systemic anticoagulation for an indication other than IHD, urea volume distribution calculated by the Watson’s formula [21], dialysis membrane surface area (2.1m^2^ or 1.8m^2^), sodium concentration of the dialysate, bicarbonate concentration of the dialysate, calcium concentration of the dialysate (1.25 or 1.50 mmol/L), invasive mechanical ventilation during the session, and vasopressor amines infusion during a session.

Several criteria of efficacy were collected; these included the following: (i) prescribed and delivered IHD duration, (ii) prescribed and delivered blood flow, (iii) prescribed and delivered dialysate flow, (iv) prescribed ultrafiltration and net ultrafiltration at end of the session, and (v) the Kt/V measured by the ionic dialysance at the end of the session. We determined the Kt/V per hour and the Kt at the end of session from the collected data, as previously stated.

Regarding safety, several criteria of safety were collected and these included the following: (i) the occurrence of hypocalcemia below 0.90 mmol/L requiring calcium infusion and the cardiac events related to RCA during the session, (ii) hemorrhagic events, (iii) interventions for hemodynamic instability, and (iv) causes of premature termination of a session, including hemodynamic instability, catheter dysfunction, acute high transmembrane pressure, and circuit clotting.

Hemorrhagic events were defined as follows [22]: Type I as a severe hemorrhagic event, such as a hemorrhagic shock and/or the need for embolization, and/or need for surgery within 24 h following an IHD session; Type II as an intermediate hemorrhagic event, such as a transfusion exceeding two red blood cells within 24 h or exceeding three red blood cells within four days following an IHD session; and Type III as a minor hemorrhagic event, such as macroscopic bleeding and/or transfusion of less than two red blood cells within 24 h following an IHD session.

Hemodynamic instability was defined by arterial hypotension with mean blood pressure < 65 mmHg, or a 10% decrease in mean blood pressure in cases of a mean blood pressure already below 65 mmHg before the IHD. Considered interventions for hemodynamic instability were ultrafiltration stopping, fluid bolus, and increasing the dose of vasopressor amines [23, 24].

Catheter dysfunction during IHD was defined as a problem with catheter flow, unfavorable inflow and outflow line pressures requiring catheter mobilization, inversion of lines, and flush through the catheter lumen [25].

### Statistical analysis

The primary objective was to show an increase in efficacy, quantified with Kt/V from the ionic dialysance at the end of an IHD session, for the RCA group compared to the heparin group. We calculated that at least 65 IHD sessions per group were necessary to show a 0.15 absolute difference of Kt/V for a two-sided type I error at 5% and a statistical power of 80%, taking into account a 0.3 standard deviation of Kt/V [26]. To account for potential inter- and intra-individual variability (some IHD sessions for the same patient), at least 80 IHD sessions per group were necessary. The secondary endpoint was to compare the safety, including cardiac events related to RCA during a session, hemorrhagic events, interventions for hemodynamic instability, and causes of premature termination of IHD session. We performed exploratory analyses on the changes in efficacy and safety between an implementation period (2015) and at long term (2017).

The population was described by the number and percentages for the categorical variables and by the mean (± standard deviation) or median [interquartile range] for quantitative variables, respectively, of their statistical distribution. Normality was examined by the Shapiro–Wilk test.

For non-repeated data (patient unit), inter-group comparisons (RCA vs. heparin, 2015 vs. 2017) were performed for quantitative variables with Student's t-test or Mann–Whitney's test if t-test conditions were not met. Homoscedasticity was assessed by the Bartlett test. Inter-group comparisons for categorical variables were performed with Chi–square test or by the Fisher exact test. The study of relationships between quantitative variables was conducted by estimating the Pearson or Spearman correlation coefficient depending on the nature of the statistical distribution. For correlated data (IHD session unit), these analyses were supplemented by mixed regression considering the patient as a random effect to consider inter- and intra-individual variability (some IHD sessions for the same patient).

Inverse probability of treatment weighting was then carried out as a propensity analysis by assigning each participant an inverse weighting of the probability of receiving or not receiving a RCA procedure. The weight of patients who were highly likely to receive RCA based on their observable characteristics was reduced and that of patients who were unlikely to receive RCA was increased. Both RCA and heparin groups were rendered comparable because they would have had the same chance of being used. The results were expressed using absolute differences and 95% confidence intervals (CI). The validity of matching was tested by analyzing the standardized differences |d| with |d|> 0.2 considered as an imbalance.

All tests were two sided and a *p*-value < 5% was considered statistically significant. Individual *p*-values were reported without applying type I correction but paying on specific attention to the magnitude of differences [27]. Analyses were performed with Stata software (Version 15, StataCorp, College Station US).

## Results

**Fig. 1:**
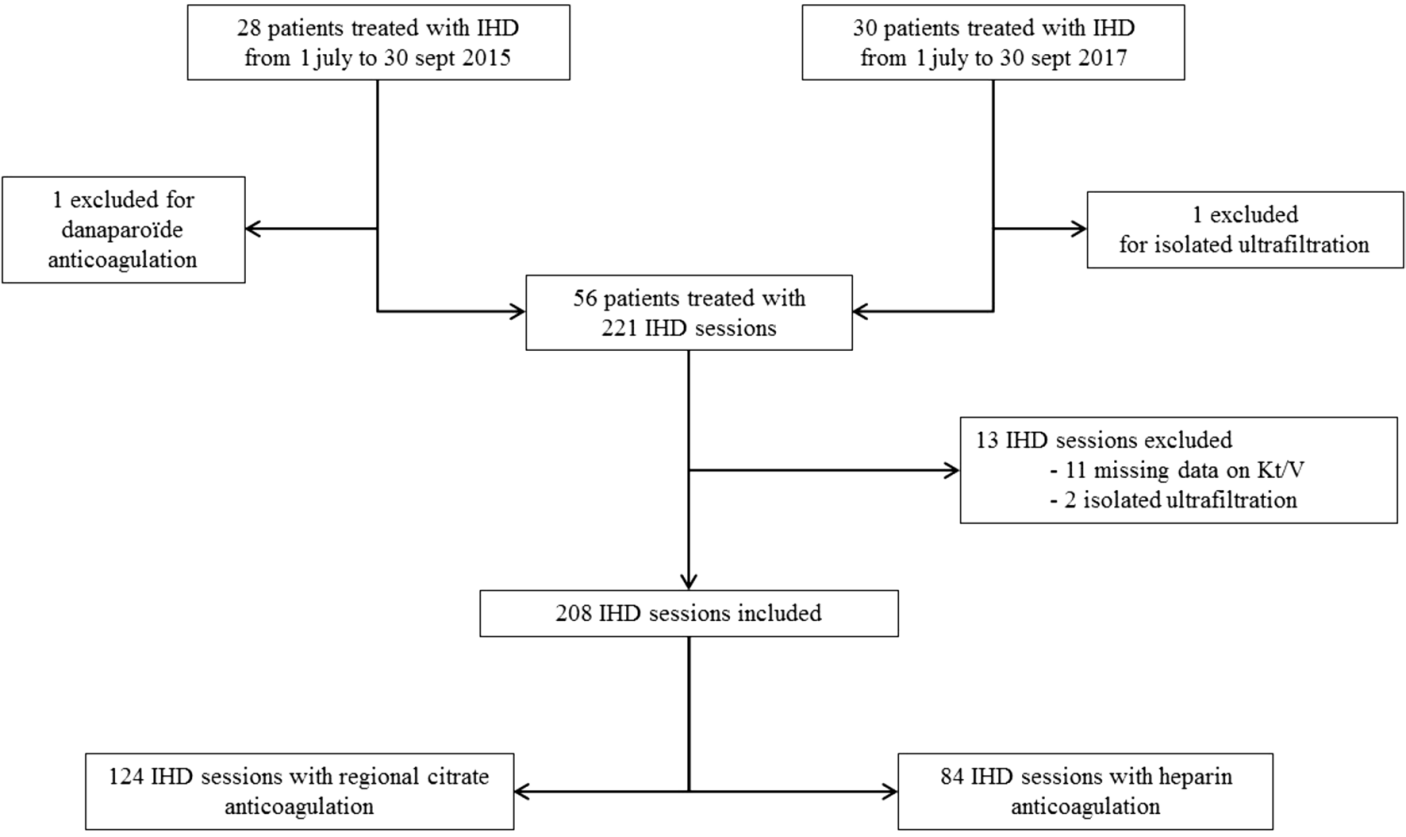
Flowchart of patients treated with intermittent hemodialysis in 2015 and 2017. IHD intermittent hemodialysis

Overall, 208 IHD were analyzed, including 124 sessions using RCA and 84 sessions using heparin anticoagulation (Fig. 1). Characteristics of patients and the IHD sessions are presented in Table 1. Characteristics for the two time periods are presented in Additional file 2: Table S1. There was a higher proportion of patients with arteriovenous fistula or tunneled catheter in the heparin group. There were also significantly fewer patients treated with vasopressor amines in the RCA group.

**Table 1:** Characteristics of the patients and IHD sessions

Variables	Before inverse probability of treatment weighting			After inverse probability of treatment weighting			
	RCA group	Heparin group	*p*-value	RCA group	Heparin group	*p*-value	|d|
Patients, *n*	40	35		38	34		
Male gender, *n* (%)	26 (65)	26 (74)	0.38	(66)	(73)	0.60	0.141
Age, years	66 ± 16	69 ± 14	0.36	67 ± 16	67 ± 16	0.97	0.013
Weight at ICU admission, kg	78.7 ± 20.2	78.9 ± 16.4	0.88	81.7 ± 19.8	76.9 ± 15.0	0.25	0.27
BMI at ICU admission, kg/m^2^	27.6 ± 6.4	28.1 ± 6.3	0.72	28.5 ± 6.3	27.1 ± 5.6	0.32	0.23
SAPS II at ICU admission, points	65 ± 22	62 ± 20	0.62	61 ± 21	63 ± 22	0.81	0.066
Sepsis in ICU stay, *n*(%)	26 (65)	24 (69)	0.74	(68)	(75)	0.56	0.151
Chronic dialysis patients, *n*(%)	9 (22.5)	9 (26)	0.75	(20.4)	(35)	0.26	0.329
IHD sessions, *n*	124	84		112	82		
Vascular access for IHD, *n*(%)						0.98	
Arteriovenous fistula	3 (2.4)	11 (13.1)	0.004	(6.8)	(6.9)		0.005
Temporary central femoral catheter	65 (52.4)	33 (39.3)	0.07	(48.4)	(44.7)		0.073
Temporary central right jugular catheter	37 (29.9)	16 (19)	0.10	(24.0)	(27.8)		0.085
Temporary central left jugular catheter	2 (1.6)	1 (1.2)	1	(1.9)	(1.4)		0.040
Tunneled central venous access	17 (13.7)	23 (27.4)	0.02	(18.9)	(19.2)		0.005
Urea volume distribution, L	36.9 ± 6.2	39.1 ± 6.2	0.17	37.5 ± 6.3	38.7 ± 6.3	0.23	0.197
Sodium concentration of the dialysate, mmol/L	139 ± 2	139 ± 2	0.99	139 ± 2	139 ± 2	0.58	0.087
Bicarbonate concentration of the dialysate, mmol/L	33 ± 3	34 ± 2	0.13	33 ± 3	34 ± 2	0.10	0.247
Dialysate with calcium concentration of 1.50 mmol/L, *n*(%)	87 (70.2)	77 (91.7)	0.001	(70.4)	(89.6)	0.008	0.495
Dialysis membrane surface are of 2.1m^2^, *n* (%)	106 (85.5)	65 (77.4)	0.064	(86.3)	(70.8)	0.01	0.386
Systemic anticoagulation for an indication other than IHD, *n*(%)	33 (26.6)	30 (35.7)	0.04	(33.7)	(39.2)	0.50	0.113
Contraindication to heparin anticoagulation, *n*(%)	16 (13)	0 (0)	0.001	(13)	(0)	0.001	0.537
Invasive mechanical ventilation during IHD, *n* (%)	46 (37)	33 (39)	0.21	(36)	(41)	0.48	0.113
Vasopressor amine infusion during IHD, *n* (%)	39 (31)	36 (43)	0.006	(29)	(42)	0.06	0.289

RCA, regional citrate anticoagulation, BMI, body mass index, SAPS, simplified acute physiology score, IHD, intermittent hemodialysis

### IHD efficacy

Concerning the primary endpoint, there was no difference between the RCA and heparin coagulation groups based on the Kt/V at the end of an IHD session (Table 2). There was also no difference found for the Kt/V per hour or Kt (Table 2). The non-significant difference on KT/V between the RCA and heparin coagulation groups was confirmed by the propensity analysis using inverse probability of treatment weighting. There was a decrease between the prescribed and delivered duration of IHD sessions in the RCA group and a decrease between the prescribed and delivered blood flow in the heparin group.

**Table 2:** Comparison in efficacy between RCA and heparin anticoagulation for IHD

Variables	Before inverse probability of treatment weighting			After inverse probability of treatment weighting			
	IHD sessions with RCA (*n* = 124)	IHD sessions with heparin (*n* = 84)	*p*-value	IHD sessions with RCA (*n* = 112)	IHD sessions with heparin (*n* = 82)	*p*-value	|d|
Prescribed duration, min	271 ± 92	252 ± 59	0.35	271 ± 94	251 ± 57	0.07	0.250
Delivered duration, min	225 ± 84	234 ± 70	0.32	228 ± 78	229 ± 71	0.91	0.017
Difference between prescribed and delivered duration, min	45 ± 97	18 ± 50	0.01	43 ± 97	22 ± 56	0.07	0.262
Prescribed blood flow, mL/min	237 ± 39	257 ± 33	0.02	243 ± 40	254 ± 31	0.06	0.296
Delivered blood flow, mL/min	232 ± 36	245 ± 31	0.11	237 ± 37	236 ± 30	0.82	0.037
Difference between prescribed and delivered blood flow, mL/min	5 ± 19	12 ± 26	0.04	6 ± 18	18 ± 28	0.003	0.533
Prescribed dialysate flow, mL/min	481 ± 59	490 ± 46	0.43	488 ± 57	484 ± 52	0.70	0.070
Delivered dialysate flow, mL/min	485 ± 52	488 ± 50	0.81	490 ± 54	482 ± 57	0.39	0.159
Prescribed ultrafiltration, L	1.19 ± 0.98	1.30 ± 0.97	0.71	1.22 ± 0.99	1.20 ± 0.98	0.92	0.016
Net ultrafiltration at the end of session, L	0.96 ± 0.92	1.13 ± 0.91	0.33	1.02 ± 0.91	1.03 ± 0.94	0.93	0.015
Difference between prescribed and net ultrafiltration, L	0.23 ± 0.51	0.17 ± 0.34	0.34	0.20 ± 0.48	0.17 ± 0.35	0.65	0.069
Kt/V per hour	0.247 ± 0.068	0.227 ± 0.063	0.08	0.243 ± 0.072	0.217 ± 0.063	0.02	0.392
Kt/V at the end of session	0.95 ± 0.38	0.89 ± 0.32	0.98	0.93 ± 0.38	0.84 ± 0.32	0.61	0.242
Kt at the end of session	33.2 ± 13.4	34.5 ± 12.7	0.58	33.7 ± 13.5	32.0 ± 12.6	0.42	0.126
Kt/V at the end of session in 2015	0.97 ± 0.37	0.86 ± 0.30	0.53	NE	NE		
Kt/V at the end of session in 2017	0.91 ± 0.41	0.92 ± 0.33	0.51	NE	NE		
Kt at the end of session in 2015	31.9 ± 10.9	31.1 ± 10.3	0.80	NE	NE		
Kt at the end of session in 2017	35.0 ± 16.1	37.3 ± 13.9	0.54	NE	NE		

NE not evaluated (Inverse probability of treatment weighting was not applied to the subgroup analyses in 2015 and 2017), *RCA* regional citrate anticoagulation, *IHD* intermittent hemodialysis

### IHD safety

Concerning the secondary endpoints, there were more occurrences of a premature termination resulting from acute high transmembrane pressure or circuit clotting in the RCA group (Table 3). Patients in the RCA group were more often required calcium recharging without an increase in the occurrence of a bleeding event. No cardiac events related to RCA occurred during IHD. RCA was associated with an increased risk of circuit clotting (absolute differences = 0.10, 95% CI [0.03–0.18]; *p* = 0.008) in the propensity analysis adjusted for vascular access, BMI, amine infusion during IHD, delivered blood flow, net ultrafiltration, sepsis, SAPS II score, and periods (2015 and 2017).

**Table 3:** Comparison in safety between RCA and heparin anticoagulation for IHD

Variables	Before inverse probability of treatment weighting			After inverse probability of treatment weighting			
	IHD sessions with RCA (*n* = 124)	IHD sessions with heparin (*n* = 84)	*p*-value	IHD sessions with RCA (*n* = 112)	IHD sessions with heparin (*n* = 82)	*p*-value	|d|
Calcium gluconate infusion for hypocalcemia during session, *n* (%)	10 (8.1)	1 (1.2)	0.008	(6.9)	(2.1)	0.22	0.235
Hemorrhagic events, *n* (%)						0.15	
Type I	0 (0)	0 (0)	1	0 (0)	0 (0)		NA
Type II	2 (1.6)	0 (0)	0.52	(2.7)	(0)		0.235
Type III	5 (4)	7 (8.3)	0.21	(3.8)	(8.9)		0.211
Interventions for hemodynamic instability, *n*(%)							
Ultrafiltration stopping	12 (9.7)	16 (19.1)	0.11	(9.0)	(17.8)	0.09	0.261
Fluid bolus	5 (4)	6 (7.1)	0.33	(3.7)	(7.1)	0.29	0.151
Increasing dose of vasopressor amine	22 (17.7)	19 (22.6)	0.08	(14.7)	(20.6)	0.29	0.155
Causes of premature termination of session, *n* (%)							
Hemodynamic instability	6 (4.8)	6 (7.1)	0.49	(4.4)	(9.1)	0.22	0.188
Catheter dysfunction	7 (5.6)	7 (8.3)	0.37	(5.5)	(11.4)	0.16	0.213
Acute high transmembrane pressure	26 (21)	6 (7)	0.02	(20.2)	(8.8)	0.03	0.327
Circuit clotting	16 (12.9)	2 (2.4)	0.02	(12.2)	(2.9)	0.02	0.355

NA, not applicable*, *RCA, regional citrate anticoagulation, IHD intermittent hemodialysis

### IHD efficacy and safety between 2015 and 2017

Regarding the exploratory analyses, no difference in efficacy and safety was found between the two study periods in 2015 and 2017 (Table 4).

**Table 4:** Comparison in efficacy and safety between the two study periods

Variables	IHD sessions in 2015 (*n* = 116)	IHD sessions in 2017 (*n* = 92)	*p*-value
IHD session with RCA, *n* (%)	76 (65.5)	48 (52.1)	0.97
Kt/V at the end of session	0.93 ± 0.35	0.92 ± 0.38	0.84
Kt/V at the end of session with RCA	0.97 ± 0.37	0.91 ± 0.41	0.72
Kt/V at the end of session with heparin	0.86 ± 0.30	0.92 ± 0.33	0.59
Causes of premature termination of session, *n*(%)			
Acute high transmembrane pressure	21(18)	11 (12)	0.23
Circuit clotting	11 (9.5)	7 (7.6)	0.63

RCA, regional citrate anticoagulation, IHD, intermittent hemodialysis

## Discussion

Our study showed that there was no difference in efficacy between RCA and heparin anticoagulation for IHD. RCA was found to be associated with an increased risk of circuit clotting resulting in premature termination of IHD session in ICU patients non-selected. Safety and efficacy of RCA at the implementation in the ICU were similar to that in the long term.

To date, two procedures using citrate for IHD are described: (i) citrate infusion connected to arterial line associated with a diffusion of calcium contained in the dialysate [14] which was the procedure used in our study; (ii) a loss of calcium by a calcium-free citrate-containing dialysate associated with calcium reinjection connected to the venous line [13, 15]. In CRRT, the RCA is performed by citrate infusion connected to arterial line associated with calcium reinjection connected to the venous line. Currently, the very high flow rates used in IHD prevent the use of this CRRT procedure for IHD.

Our study is, to our knowledge, the first to compare the efficacy of RCA to heparin anticoagulation for IHD in ICU patients. There are limited data on the efficacy of RCA for IHD. In 2017, Robert et al. assessed the efficacy over the duration of the IHD session. They reported that 25 out of 29 IHD sessions were completed within the prescribed 240 min [15]. In our study, we choose the dialysis dose as the efficacy endpoint, estimated through the Kt/V. Despite several limitations, Kt/V is the gold standard for the dialysis dose endpoint and used in major randomized trials [26, 28]. Faguer et al. reported, in 2017, a series of 101 IHD sessions using RCA with an average Kt/V, calculated by the log (predialysis urea/postdialysis urea) formula, of 1.1 ± 0.4 [13]. This Kt/V is higher than the results found in our study. This could be explained by a different Kt/V estimation method. The KT/V in our study was measured from ionic dialysance, which is currently the reference method for quantifying Kt/V in IHD [29, 30]. Furthermore, the median of the session durations and the blood flow (300 ml/min) in the Faguer et al. study were higher than in our study. These IHD modalities were made possible as more than 40% of patients had a vascular access for chronic dialysis, and few patients undergoing vasopressor treatment [13]. Further studies need to be conducted to compare the efficacy and safety of the different procedures using citrate anticoagulation for IHD.

Our study also assessed the safety of RCA during IHD. We used a RCA procedure previously reported by Fiaccadori et al. [14] and did not observe a severe event but an increase in clotting circuit. In the Fiaccadori et al. study, in which the majority of patient was a surgical ICU population, the calcium concentration in the dialysate was of 1.25 mmol/L while a dialysate with calcium concentration of 1.5 mmol/L was mostly used in our study which could have led to a rapid correction of blood calcium levels resulting in clotting. We reported a higher rate of IHD session requiring a calcium infusion for hypocalcemia (8.1%) than their study (3.4%) that did not support the widespread use of a dialysate with a calcium concentration of 1.25 mmol/L in medical ICU patients. However, our results lead to imagine an infusion of calcium without increasing the circuit clotting. The use of a dialysate with calcium concentration of 1.25 mmol/L associated with additional calcium infusion with another line connected to venous line could be assessed in future study.

The RCA method has been implemented in our ICU with a short learning curve leading to a satisfactory efficacy and safety for routine clinical practice. No case of life-threatening hypocalcemia or acid–base disturbances was observed. This result suggests the possibility for the adoption of RCA for IHD at the ICU. In our study, when a patient had a contraindication to heparin anticoagulation, RCA was consistently chosen, probably to decrease the risk of bleeding or any other complications related to heparin. The use of RCA for IHD could be an innovative solution in ICU patients mainly when they have acute heparin-induced thrombocytopenia syndrome or high risk of bleeding or acute bleeding [31].

Our study encountered several limitations. Firstly, the retrospective study design may have led to selection biases that could have impacted the results. To reduce selection biases, inclusion criteria were made broad to allow the analysis of all patients during the study period. The retrospective design, however, had the advantage of substantially reducing the exclusion criteria compared with a randomized controlled trial and therefore being fully applicable to daily clinical practice. Secondly, our study was not designed to identify a difference in the safety endpoints and may be underpowered to identify differences between RCA and heparin during IHD. Thirdly, RCA could have impacted the Kt/V measurements by ionic dialysance. No data were found to support this hypothesis and the Kt/V values found in our study were in range reported in the literature. Lastly, it is possible that the increased risk of circuit clotting is the consequence of factors other than relative RCA failure. A propensity analysis was performed to consider the confounding factors.

## Conclusion

The use of RCA with calcium infusion by diffusive influx from dialysate for IHD sessions could be a simple method to implement in an ICU with stable long-term efficacy and safety. There was no statistical difference found in the efficacy of RCA compared to heparin anticoagulation. This RCA procedure, however, could be associated with an increased risk of circuit clotting compared to heparin anticoagulation in non-selected ICU patients. Other differences of uncommon safety endpoints may not have been identified in our study because of a lack of statistical power. Further studies need to be conducted to define the best anticoagulation for IHD in ICU and the role of RCA for IHD in ICU patients.

## Supplementary Information

**Additional file 1**: **Figure S1.** Schematic representation of regional citrate anticoagulation for intermittent hemodialysis.

**Additional file 2**: **Table S1.** Characteristics of patients in the two study periods.

## Data Availability

The data that support the findings of this study are available from the corresponding author upon reasonable request.

## Abbreviations

BMI: Body Mass Index
CI: Confidence Intervals
CRRT: Continuous renal replacement therapy
ICU: Intensive Care Unit
IHD: Intermittent Hemodialysis
RCA: Regional Citrate Anticoagulation
RRT: Renal replacement therapy
SAPS: Simplified Acute Physiology Score
