# Supplementary figures and images for: Comparison between regional citrate anticoagulation and heparin for intermittent hemodialysis in ICU patients: a propensity score-matched cohort study
Source: Ann Intensive Care. 2021 Jan 22;11:13. doi: 10.1186/s13613-021-00803-x (PMC7822996; doi:10.1186/s13613-021-00803-x)

**Additional files**

**Additional file 1**

**Figure S1**

**
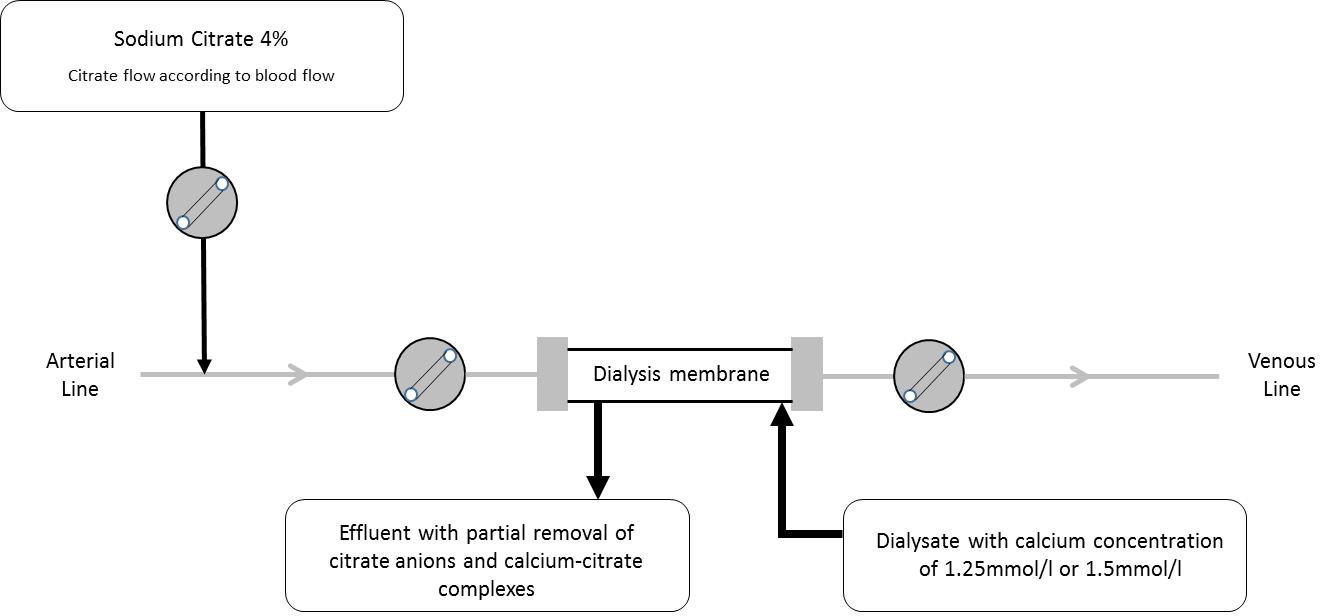
**

Supplement: Supplementary file 1 — Additional file 1: Figure S1. Schematic representation of regional citrate anticoagulation for intermittent hemodialysis. [file 13613_2021_803_MOESM1_ESM.docx]
