# Supplementary material for: Comparison between regional citrate anticoagulation and heparin for intermittent hemodialysis in ICU patients: a propensity score-matched cohort study
Source: Ann Intensive Care. 2021 Jan 22;11:13. doi: 10.1186/s13613-021-00803-x (PMC7822996; doi:10.1186/s13613-021-00803-x)
Supplement: Supplementary file 2 — Additional file 2: Table S1. Characteristics of patients in the two study periods. [file 13613_2021_803_MOESM2_ESM.docx]

**Additional file 2**

**Tableau S1:** Characteristics of patients in the two study periods

|  | 2015  (n=27) | 2017  (n=29) | p-value |
| --- | --- | --- | --- |
| Male gender, n (%) | 17 (63) | 19 (66) | 0.84 |
| Age, years | 67 ± 19 | 66 ± 12 | 0.77 |
| Weight at ICU admission, kg | 76.4 ± 15.7 | 80.3 ± 22.3 | 0.93 |
| BMI at ICU admission, kg/m² | 27.9 ± 6.7 | 27.7 ± 6.6 | 0.60 |
| SAPS II at ICU admission, points | 68 ± 24 | 60 ± 19 | 0.26 |
| Sepsis in ICU stay, n (%) | 13 (48) | 23 (79) | 0.02 |
| Chronic dialysis patients, n (%) | 5 (19) | 8 (28) | 0.53 |
| Number of IHD sessions per patient | 3 [1-6] | 2 [2-4] | 0.49 |

*RCA* regional citrate anticoagulation, *BMI* body mass index, *SAPS* simplified acute physiology score
